# Boundaries Between Research Ethics and Ethical Research Use in Artificial Intelligence Health Research

**DOI:** 10.1177/15562646211002744

**Published:** 2021-03-18

**Authors:** Gabrielle Samuel, Jenn Chubb, Gemma Derrick

**Affiliations:** 1 Department of Global Health & Social Medicine, 4616 King’s College London, London, UK; 2 Department of Computer Science, 8748 University of York, Heslington, York, UK; 3 Department of Educational Research, University of Lancaster, Lancaster, UK

**Keywords:** responsible research, artificial intelligence, ethics, research ethics, privacy, health, responsible research and innovation, societal impact

## Abstract

The governance of ethically acceptable research in higher education institutions has been under scrutiny over the past half a century. Concomitantly, recently, decision makers have required researchers to acknowledge the societal impact of their research, as well as anticipate and respond to ethical dimensions of this societal impact through responsible research and innovation principles. Using artificial intelligence population health research in the United Kingdom and Canada as a case study, we combine a mapping study of journal publications with 18 interviews with researchers to explore how the ethical dimensions associated with this societal impact are incorporated into research agendas. Researchers separated the ethical responsibility of their research with its societal impact. We discuss the implications for both researchers and actors across the Ethics Ecosystem.

## Introduction

The governance of ethically acceptable research behavior in higher education institutions (HEIs) has been under close scrutiny over the past half a century following a series of atrocities relating to a misuse of human participants (Boden et al., 2009; Stark, 2011; Truman, 2003). This reflects a desire to normalize personal ethical barometers regarding how to conduct human participant research, specifically in relation to respect for persons, justice, and beneficence. Behaving “ethically” in human participant research is reinforced through the “Ethics Ecosystem” (Samuel et al., 2019)—an evaluation network of interconnected actors existing at a number of levels within the academic system and at different points of the research production process. This notion comprises individuals (researchers), organizations (research institutions and the various committees within, such as research ethics committees [RECs] in the United Kingdom; also called research ethics boards [REBs] in Canada or institutional review boards [IRBs] in the United States), and external bodies (publishing houses, funding bodies, professional associations, and the governance policies they produce) who participate equally in the promotion, evaluation, and reenforcement of a shared understanding of responsible research behavior (Samuel et al., 2019).

Alongside this, formal recognition of the societal impact of research beyond academia is gaining prominence as an embedded principle associated with knowledge production and research excellence (Derrick, 2018). The inclusion of impact as a criterion in research funding and assessment—for both impact generation and for the purposes of evaluating research quality—formally ensures that engagement with and ownership of impact outcomes become desirable academic behaviors. This aims to incentivize researchers to ensure their research responds to local and global challenges, and provides a level of accountability to decision makers. Notwithstanding the politicization of the impact agenda (Deem et al., 2008; Research Council Economic Impact Group, 2006; Watermeyer, 2019), the move toward demonstrating impact can be seen to appeal to academics’ intrinsic responsibilities, where the desire to generate research impact and contribute toward society (Chubb, 2017) contributes to a long-standing vision that academics *ought* to locate a sense of responsibility to communicate their work to the public and others (Bodmer, 1986; Chubb, 2017; Chubb & Reed, 2017; Weinstein et al., 2021).

Furthermore, while research evaluation practices are cementing the need for researchers to consider the societal impact of their work, the science and technology field is concurrently bringing societal impact into the research process through responsible research and innovation (RRI), representing a move toward a “science *for* and *with* society” (Laroches, 2011; cited in Owen & Macnaghten, 2012). Although debates exist about how to effectively define RRI, all permutations of the concept converge around the need for responsibility and integrity to be embedded into research governance, and that RRI should be a collective endeavor to anticipate and respond to the ethical, social, and political dimensions associated with the societal impact of research across all stakeholders (Stilgoe et al., 2013).

### The **“**Digital Turn,” Artificial Intelligence and Ethics

With the advent of the “digital turn,” traditional research methods have been reimagined, with new forms of data available to study (social media, blogs, data from wearable devices, and electronic health records) and new methodological tools to help researchers to access, process, and harness this data (artificial intelligence [AI], data modeling). This has presented specific ethical challenges, and in some instances, has disrupted “traditional” understandings of research ethics, problematizing notions of consent and privacy, and raising questions around what constitutes human participant research (we prefer the notion “data subjects”; Ess, 2017; Samuel et al., 2018; Whiteman, 2012). In fact, digital research ethics has been described as one of flux and uncertainty as the new boundaries of what is ethical are still being debated and tested (Metcalf et al., 2016; Whiting & Pritchard, 2017).

In particular, the ethical implications of AI have received much attention in recent years. We refer to AI as “the capability of a machine to imitate intelligent human behavior” (Peters et al., 2020). AI is, of course, ubiquitous—applied in a range of research arenas, settings, and domains of use. To consider the impacts of AI as homogenous is therefore somewhat unhelpful. Instead, AI is an umbrella term, or a “collection” of technologies with wide-ranging impacts, benefits, and challenges. Although dominant narratives often associate AI with anthropomorphized “scary robots” and the world of science fiction (Cave et al., 2020), it is often when we look to the present and real-world applications that we find AI is at the heart of social, cultural, and economic impact in the world, both in terms of our every day (the use of smartphones, AI voice assistance, search algorithms, and AI in games) and the more spectacular (computer–human interfaces, self-driving cars, etc.). Indeed, now so more than ever, AI and its impacts have come into sharp focus during the 2020 COVID-19 pandemic, which saw the role of AI becoming a focal point of discussion (Vaishya et al., 2020). Here, scholars have emphasized the urgency to consider the use of AI tools for diagnosis, prognosis, and containment of the coronavirus, as well as to consider the associated ethical implications (Tzachor et al., 2020). Such ethical implications (specifically and more generally) include, among others, matters of public and stakeholder trust in AI systems, the need for accountability of the technology, responsibility, and transparency and explainability of algorithms. Questions about privacy in terms of data subjects and fairness and justice regarding access to AI technology are also prominent (Blasimme & Vayena, 2019; Cath et al., 2018; Dignum, 2018; Vayena et al., 2018; Vollmer et al., 2020) as are concerns about bias in data sets (Ledford, 2019; Noor, 2020; Parikh et al., 2019).

### AI, Ethical Governance, and Research Practice

Presently, regulators, industry,^1^ and scholars are working on ways to ensure AI is designed, developed, and introduced at the societal level responsibly, and research institutes focused on the future of AI are strategically trying to ensure that the ethics of AI and related systems in a range of settings are robustly studied and scrutinized.^2^ There has been a plethora of published international and national guidelines, recommendations, statements, and documents related to AI ethics. In fact, to date, over 80 such “AI ethics” initiatives have published reports describing high-level ethical principles, tenets, values, or other abstract requirements for AI development and deployment as organizations and institutions start to grapple with how best to govern AI research and use (Jobin et al., 2019). Despite the “ethicization of AI,” and the concerted effort to embed ethical thinking and governance into the research and development of AI, less is known about the research ethics processes that involve the use of AI and how much they consider both research ethics issues as well as the ethics of research use. This is important given the calls for RRI, and for researchers to anticipate and respond to the ethical dimensions associated with the societal impact of research. It is vital to understand how researchers construct any responsibilities associated with the ethics of the societal impact of their research, and whether ethics governance frameworks exist to support such responsibilities. Mittelstadt (2019) notes that there is a lack of empirical evidence linking codes of best practice ethics to actual impact on AI researcher behavior, unless they are developed alongside structures of accountability and workplace practices. This is compounded by AI ethics guidelines being described as “vague, high-level principles, and value statements” (Mittelstadt, 2019, p. 1) that provide few practical recommendations for AI researchers (Mulgan, 2019).

Our aim was to empirically explore the extent to which AI researchers consider research ethics in their work, and how far, if at all, this extends to the consideration of the ethics associated with the broader, societal impact of research. As researchers interested in health, our case study was the field of population health research that uses AI methods. Our definition of “researcher,” and to reflect a more inclusive consideration of “value” beyond the mainstream academic community, was broadened to include anyone involved in AI research. This was particularly important as, between different institutional contexts, the idea of ethical AI research practice is not always shared. This offers a useful case study: health research is often governed by strict ethics governance requirements at both the research and societal impact level, however ethics governance structures associated with population health research are blurry, this can also mean between different types of public organizations involved in research, such as universities, hospitals, and other health stakeholders. Population health research using clinical data and/or having clinical applications (e.g., research into cancer prediction, and diagnosis research for heart disease, eye disease, stroke, Alzheimer's disease, and depression) will fall under strong research (REC/REB) and professional/health sector ethical governance remits. Research using nonclinical data, however, may or may not be under the remit of RECs/REBs; similarly, nonclinical applications of population health AI research are unlikely to fall under the regulatory structures of the clinical sector. This may include, for example, researchers who are exploring how AI systems can help predict epidemics and disease outbreaks (Bengtsson et al., 2015; Harris et al., 2017; Naghavi et al., 2010), as well as various health and mental health states (for a review, see, e.g., Blasimme & Vayena, 2019). Understanding ethics decision making in this space, and how researchers engage with both research ethics, as well as the ethics of research use, can help expose inconsistencies or issues with current practices and/or governance structures.

We drew on a mapping study of publications reporting AI population health-related research, and semistructured interviews with AI population health-related researchers from the United Kingdom and English-speaking Canada (*n* = 18). Our research questions were (a) what are interviewees’ concerns and considerations pertaining to the ethical decision-making responsibilities they assigned to themselves, as well as other actors in terms of their research and its potential impact and (b) using coauthorship mapping, how are nonacademic health stakeholder organizations represented in university–stakeholder partnerships in research production, and how do these different actors perceive AI research ethics differently. Although the specific types of research required to be reviewed by an REC varies per country, the United Kingdom and Canada have similar research ethics governance structures (including their RECs and REBs), both represent hubs for “ethical AI” discussions and practice (Malone, 2019; Sloane, 2018), and investment in AI places them only behind China and the United States.^3,4^ Our aim was not a comparative study between the United Kingdom and Canada, but rather to gain diverse perspectives about ethics decision making in the field of AI/population health research in those countries already considering ethics in policy-level discussions, to understand how (much) these discussions, if at all, become embedded into researchers behaviors and practices.

## Methods

### Interviews

#### Recruitment

Sampling was purposive. U.K. researchers were identified via an in-depth bibliometric analysis. A combined keyword-based search strategy using words and phrases commonly associated with AI and health were combined, and verified through a manual exercise by GD and GS. Prior examples of this approach are described in more detail in Samuel et al. (2019). Individual articles were then manually checked and cleaned (*n* = 244 cleaned to *n* = 58) according to the project-specific definition of “AI population health-related research.” From this, a list of relevant researchers was generated and invited to participate. Following email invitations, 10 researchers agreed to participate. Bibliometric sampling was not required for Canadian interviewees; rather the Canadian Institute of Health Research (CIHR) provided an initial list of researchers in the relevant research area, and this was followed up with snowball sampling. Fifteen researchers were invited for interview and eight researchers agreed to participate. This higher response rate was likely attributed to differences in recruitment method, though could also have led to biases in the data set because of the reliance of the CIHR as a gatekeeper.

#### Demographics

The sample comprised mainly male participants (*n* = 14/18; in line with heavy bias in the field [Leavy, 2018]). Interviewees were from a range of seniority levels (eight professors, research chairs, or heads of research teams; seven associate/assistant professors or lectures/senior lecturers; two research fellows/associates; one PhD); from 14 different universities (nine United Kingdom and five Canadian); working with a range of data (clinical, health survey, and user sensor); and from a range of disciplines, including computer science, informatics, data science, epidemiology, public health, and statistics. Some interviewees identified themselves as working across two disciplinary domains: *n* = 8 positioned themselves as computer scientists, *n* = 8 positioned themselves as population health, statistics, and/or epidemiology experts, and *n* = 6 positioned themselves as data scientists and/or informaticians.

Our interviewees were self-selected, which may bias our research data. However, it was not our desire to achieve a representative sample of interviewees, rather, and as per the nature of exploratory research, we were interested in interviewing a purposive sample.

#### Data Collection and Analysis

Interviews were conducted by GS and JC, either face to face, or via Skype/phone, and were audiorecorded. Interviews explored each interviewee's perceptions on the ethical issues surrounding AI population health research, including their own experiences around this; as well as their views and experiences of decision making around ethical approval processes. Analysis of interview data was inductive and completed in two interlinked rounds: broad coding (memo making and scanning interview transcripts for relevant themes) was performed independently by GS and JD. Themes were then discussed to check similarities. A detailed coding was then performed by GS using Nvivo software (Strauss, 1987). Given the small sample size, distinctions were not made between different interviewees’ disciplines or country of residence during analysis. Where differences between the U.K. and Canadian interviewees were present, these are noted in the findings. The study received ethics clearance from King's College Research Ethics Committee (MRM-18/19-10499).

#### Limitations

First, our research approach is qualitative and exploratory, and as such, does not require a representative sample size to rigorously analyze the data. Having said that, we do note that the small sample size makes it difficult to make generalizations about the findings. Second, the self-selection of participants is not necessarily problematic for exploratory research as it allows the exposure of key themes, although it does bias the sample, and this needs to be considered in drawing too strong conclusions from the findings. Finally, adopting a case study approach does not permit us to make generalizations about our findings beyond population health AI research, although as an exploratory piece of work, it does allow for any findings to inform future research in other areas.

### Mapping

To examine how health stakeholder organizations are represented in university–stakeholder partnerships in research production, all publications (*n* = 394) from the initial bibliometric exercise described above (see sampling) were used to discern the organizational affiliations of all contributing authors in the United Kingdom. VOSViewer 1.6.14. was used to construct coauthorship relations’ networks visualized as affiliation names to specify organizations that were either university, hospital, or other.

The resulting research clusters were visualized and analyzed using measures of centrality with relations within the network normalized and thresholds of 10 publications per affiliation with at least 10 citations applied in order to visualize main relations between research clusters and the affiliations of contributing authors.

## Findings

All interviewees were reflective of the fact that AI-related technologies raised a variety of ethical issues. Interviewees’ reflections on ethics and their own ethical decision-making responsibilities, implicitly or explicitly divided into two categories. On the one hand, they spoke about routine procedural research ethics issues that were typically seen to fall under HEI research ethics governance structures, and which related to interviewees’ own practices, perceived responsibilities, and decision making. On the other hand, they spoke about broader ethical issues that related more to the application of AI systems in society (ethics at the level of societal impact). In the following, we discuss these two categories in detail.

### The Research Process: Research Ethics is Synonymous With Data Governance

When asked about their own AI research ethics decision making, all interviewees spoke extensively, and at times, exclusively, about generalist issues relating to data governance. Their discussions focused on a range of concerns relating to protecting data subjects’ privacy when using data sets; ensuring data collection was restricted to only that which was required (data minimization); ensuring research data was used and stored appropriately and safely; and, where necessary, considering issues of consent to use the data. For instance, most interviewees’ research (*n* = 16) involved at least some secondary data analysis on already curated data sets. The potential for reidentification of individuals from these data sets was particularly discussed by interviewees, and data privacy was something all interviewees—no matter how they collected their data—had thought about in detail (“*we might actually be involving some kind of medical data…and some people, they don't want to let the other people know…their health status”* [interviewee 9]). This focus on data governance issues went further: interviewees explained how curators of many public health and medical data sets had already established data governance procedures in place, including strict access policies to ensure data remained confidential and safeguarded from potential misuse, and that this, for them, was enough to perceive ethics issues as being addressed. For example, when asked about their ethics decision making when using AI methods, interviewee 4 described *only* the frameworks and mechanisms pertinent to accessing a specific data set they required for their research:

it's quite easy to identify people even if the data has been de-identified so I am not able to add anything to that data. Somebody has to look at everything…if I want to load up a set of codes to run against that data, somebody checks that… I can't share it [the data] with anybody who doesn't have permission…if I want to generate any reports…again, there is a secure control process…to ensure privacy and confidentiality.

In fact, interviewees often constructed their discussions about ethics, and their perceived responsibilities thereof, solely in terms of the narrow focus of data governance, and for some interviewees, research ethics in AI *were* data governance issues (“*I think a lot of this to-do about ethics and AI, I think it's great, but I think, really, it ought to be ethics and data”* [interviewee 3]). The way in which interviewees synonymously constructed AI research ethics issues as “big data” governance issues was particularly apparent in their responses to questions that directly asked about the ethical concerns they considered when using AI methods. Interviewee 4 replied:

it's [ethics issues associated with AI methods] the idea that the research we do is very data intensive…how people use data, what the use of that data is…what are the concerns, what are the issues around governance and privacy and things like that.

Similarly, interviewee 1 responded by explaining that because their data is anonymized, ethical issues had already been addressed and they had no further responsibilities: *“currently I haven't got serious ethical concerns, mainly because the data I used is anonymised.”* Interviewee 10 reacted by speaking only about the responsibility of respecting the signed licensing agreements between themselves and data curators put in place to ensure responsible data use and data minimization: “*in terms of what I do, so obviously there is the licensing agreement… things like not then sharing those even…in a publication where it would be better to include examples of the real data.”* Of course, interviewees did mention other ethical issues that they thought about, though to a much lesser extent. For example, just over half of interviewees mentioned issues of data bias, and a small number of interviewees discussed the lack of transparency in AI research^5^ which, while increasingly important in the global ethics arena, was viewed as “not there yet” in the health sector, still suffering from “black box models” making it difficult to understand how predictions were arrived at (interviewee 2). Consent was also mentioned by a small number of interviewees, particularly by those interviewees who collected and analyzed “user” data from mobile devices.

### Data Governance and the Role of the REC

Interviewees’ predominant focus on data governance as a way to ensure responsible AI research ethics seemed to stem, at least in part, from the types of considerations they perceived REC members looked for when reviewing research proposals:

Interviewer: What are the sorts of main ethical issues that would be related to your research projects?

Interviewee 1: It mainly involves the data itself. Because our data is anonymised so the ethical committee will assess the sort of data and any harm to the patient, yes this [sic] is the sort of issue.

Interviewee 9 explained that when reviewing their AI-associated research ethics applications, REC members focus more on questions of data privacy than other ethical issues, such as those related to the research and the research findings:

they’re [the RECs are] more concerned about the privacy, it's more about data privacy. It's not about the results itself…[..].. the main issue, if the data is anonymised then they are quite happy we apply the AI approach to the data analytics.

Only two interviewees talked about how RECs have requested information on the AI software. Other interviewees painted a similar picture of how data governance issues were a centralized focus when talking about their interactions with their own RECs:

the actual algorithm, that was not, no, they [the REC] did not mention [that] (interviewee 14);

certainly in terms of how the data is handled that's a big piece [the REC ask for], the consent piece, but the AI piece, no, and I don’t think anyone has ever asked for that (interviewee 17); and

the issue [RECs have] is around data access and not about the software very much (interviewee 12).

We can propose then, that it was through the practices of the RECs, that interviewees’ perceptions of “acting responsibly” in terms of their AI research became synonymous with data governance issues. Interestingly, interviewees’ views about AI ethics—that is, perceiving AI research ethics’ issues as synonymous with data governance—were reflected in their perceptions about the appropriateness of RECs as an ethical governance layer for HEI AI research. While most interviewees acknowledged a lack of AI-specific expertise on ethics review committees (“*those ethics boards often…they are not terribly sophisticated in some of the methods to really understand the application”* [interviewee 13]), for many, this lack of expertise was unproblematic because, for them, the ethical issues of their AI research were nonexceptional compared to other ethics issues raised by “big data” more generally. Moreover, as we go on to describe below, these interviewees separated the review of research ethics practices, which they believed did not require expertise in AI methods (given the data governance focus of their perceptions of research ethics), from the oversight of ethics practices related to research use. The latter was perceived to require an understanding of AI systems because of its association with decision making, though this was not viewed to be under the purview of RECs:

people are intimidated by it [AI] and they are just “well, this is the computer stuff and we will focus on the things we understand.” Is it problematic? Broadly, yes in the sense that you would want your IRB [REC] to be fully informed of the whole package that is being proposed. In practice probably rarely: the difference between algorithm A and algorithm B is probably not the kind of thing that an ethics board really needs to be worried about unless it's being tied into some kind of decision-making…I have already said that's probably outside an IRBs remit anyway (interviewee 17)

Only some interviewees viewed this lack of expertise as problematic, and described how the ethics review process was failing to responsibly apply appropriate ethical scrutiny:

people don’t fully understand these techniques [AI], sometimes they will be “oh, OK, I will let it go because it seems OK”. But they might not be given the same level of scrutiny that more conventional studies are (interviewee 16).

For these particular interviewees, this lack of scrutiny was perceived to be associated with the lack of clarity over the types of harms that could be caused by AI research: “*there is a potential for us to harm a whole bunch of people at once even whole sub-populations could be stigmatised. But those harms seem a little less clear”* (interviewee 13). It was also perceived to be associated with the faculty in which RECs were housed. Interviewees described that in some instances, when RECs are based in medical faculties, REC members are better equipped to protect individuals from consequential physical harm from a research study compared with any potential downstream harm emerging from AI-associated research. Interviewee 17 explained:

the first time there was a joint meeting of the medical and behavioural IRBs to look at our…proposal…the behavioural people were a little bit nervous, it was very much a big brother kind of vibe to it…and the medical people were like, “wait, no-one is getting an experimental drug, there is no risk of medical complications, I don’t see a problem”.

Overall, interviewees’ views about their perceived responsibilities associated with AI ethics and data governance seemed to emanate from the practices of RECs. These views were then reflected in their positive perceptions about the appropriateness of RECs as an ethics governance layer for research that uses AI research methods.

### Societal Application of AI Systems

While interviewees viewed AI research ethics as synonymous with data governance issues, this was distinguished from the types of ethical issues interviewees’ perceived to be raised by the societal use of their research, which were considered more AI specific, and “a bit different” from the ethical issues associated with big data (research) in general: “*is AI different at the institution committee? I guess not. At the global level, yes, I think AI is a bit different”* (interviewee 16). Ethical concerns around the societal applications of AI systems dominated interview discussions much more heavily than those associated with the research process, and were considered to be more problematic:

usually people really start getting upset about the ethics—it's not at the upstream portion which I’m typically dealing with—the measured analysis of data. But in making actionable public health decisions based on that data using AI directly (interviewee 17).

These concerns predominantly revolved around AI systems’ complex and predictive nature, and to the seemingly increased authority being given to AI-type decision making in the health sector. Interviewee 8 worried about patient and public safety:

what we see emerging at the moment is delegating more authority for making decisions [in healthcare] to what might be termed AI…how do we know that machines are safely making decisions. How do we know that these machines work, because they’re generally quite different to a human judgement.

In fact, interviewees were also anxious about the risk to public and patient safety when AI systems did not make accurate predictions (interviewee 6) (something interviewee 15 explained could also happen when using predictive algorithms more generally). Interviewee 10 drew on an example of some AI software they developed to simplify the process of disseminating health information to patients, but which ended up inadvertently removing critical information. Given this, interviewees called for stakeholder education about the capabilities of, and uncertainties attached to such systems (Samuel et al., 2020). Finally, interviewees’ raised concerns around questions of ownership, agency, safety, and responsibility (Porter et al., 2018) (“*who owns the algorithm and who owns the data?…Who is responsible [if something goes wrong]”* [interviewee 2]; “*what does accountability look like?”* [interviewee 1]). Interviewee 17 explained:

like if a human agent which truly has agency makes a decision about a public health policy…then you can…say this person is operating in an ethical framework…But when an algorithm…makes a decision about…which town should get Ebola vaccines…to stop outbreaks…there is no clear way of attaching ethics to that decision. It's just hidden somewhere in the background in some assumption that the programmer made when they did the algorithm and they may not even realise it.

### Societal Impact of AI—The Need for Standards in Population Health

While interviewees spoke about the responsibility to ensure “downstream” (interviewee 17) concerns related to their AI-related research were addressed, these issues were generally perceived as more uncertain than research ethics issues; more difficult to manage; and, as discussed above, outside the responsibility of routine, procedural HEI research ethics governance frameworks that are overseen by RECs whose focus was perceived to be very much on concerns related to the use of AI approaches in research practice only. Interviewee 6 explicitly articulated the division that was implicitly evident in many interviewees’ narratives between research and societal impact ethics: research ethics was procedural, understood, and had a well-established governance framework, societal ethics had less formal governance structures and “fuzzy aspects”: *“there is the formal process which checks that as well. But then there is the more, let's say, less formal aspects, more fuzzy aspects, of what are possible consequences if this study is conducted.”*

Many interviewees described the lack of standards and regulation for governing AI at the level of societal impact (“*the way that ethics committees in institutions are working is still OK. But I think there needs another level of thinking that puts everything together and doesn't look at one project at a time”* [interviewee 16]). Those working in the clinical setting could and did defer to the current U.K. or Canadian clinical standards and governing norms: one U.K. interviewee referred to the well-established five tests of any healthcare intervention—safety, efficacy, cost-effectiveness, acceptability, and equity (Nilsen & Birken, 2020) that they believed were both appropriate and sufficient to ensure the responsible implementation of AI software:

Normally if we look at any kind of healthcare intervention it goes back to, I think, the five tests, which are is it safe, is it efficient, is it effective, is it acceptable, and is it equitable. And I don't see a need to change those five just for AI. I think that applies to any kind of intervention we make in healthcare (interviewee 8).

However, nearly all interviewees—both United Kingdom and Canadian—were unaware of similar governing norms and/or regulations for population health-related applications outside of the clinical realm:

at a public health level, there really is no regulation model, most of the technology they use in population health has no, at least in Canada…regulatory system for it. It's only really if you are touching individual patients in a clinical context where regulation comes in (interviewee 13).

This was problematic for many interviewees, and a consensus emerged about the perceived value of such an oversight system for the nonclinical health sector:

there definitely should be an outside body testing the models to validate. Because you can imagine, people are quite motivated to have their models used. So having…somebody validate the models. I think that would be excellent (interviewee 14)

In fact, a number of interviewees (*n* = 4) viewed this need for governance as analogous to when drug regulation was initially introduced in the pharmaceutical industry, albeit with an awareness of the shortcomings of these oversight mechanisms. In the extracts below, interviewee 6 compares the historical need for drug regulation to combat the use of ineffective and harmful drugs to stress their view about the need for better AI oversight systems. Meanwhile, interviewee 13 points to the inadequacies of drug regulation to emphasize the poor regulatory fit of drug regulation for AI systems, and the need to improve on this:

when you come up with a new drug…there's strong regulation that prevents marketing a drug that is ineffective or perhaps even harmful…but when you’re applying AI…there are not the same requirements..[so]..there is a risk to run into the same problems that have been historical problems in the development and the marketing of drugs maybe a hundred years ago (interviewee 6)

[a drug is only] licensed for a particular indication and a particular set of populations, [similarly AI systems are only developed on specific datasets], and once it's in the market it can be used by clinicians for any indication and any population..[..]..so bad things happen (interviewee 13)

It was perhaps because of this perceived lack of oversight for AI-related systems that all interviewees, when asked, narrated a sense of responsibility (“*a huge responsibility”* [interviewee 16]) to ensure the appropriate societal application of their own research. When interviewees were asked about what responsibility looked like to them, they spoke about ensuring their AI system had been validated (“*it's something you have just got to be sure it works…it's the same with anything*—*you need to make sure…they have gone through the right validation”* [interviewee 4]; that it was explainable (“*we don't use deep learning at all basically because, well because it's not explainable”* [interviewee 11]); and that it was open source (“*I would call it the open science framework…we publish the algorithm, how we created the algorithm, we publish validation data…calibration data and methods for calibrating”* [interviewee 18]). At the same time, interviewees explained the difficulties in always enacting such responsibilities. In fact, it became apparent from interviewees’ discussions that while the lack of oversight mechanisms for AI-related systems was associated, at least in part, with interviewees feeling responsibility for the impact of their research (because no other individual or institution took on this responsibility), at the same time, it was the lack of oversight that stopped interviewees enacting these responsibilities because without clear oversight infrastructures, there were no incentives to promote these responsibilities. Interviewee 6 explained:

it's easy to say “oh I think we have a responsibility” but if there are no mechanisms that at least incentivise taking responsibility then, you know, there is not much substance to such a statement….[how is] taking this responsibility…actually rewarded..[..].. if it's voluntary then no-one will do it.

In fact, interviewees provided instances of this, and of different actors in the Ethics Ecosystem who were failing to incentivize these responsibilities attached to AI ethics best practice in the promotion of AI research. Interviewee 14, for example, highlighted how journals had different requirements regarding whether AI research needed to be validated at the point of publication. Validation is an important step in the development of AI models and effective validation will ensure that models are tested with a good (ethically appropriate) representative set of input data. This testing and model validation play a necessary role in governance processes and places a responsibility on the validators to test the model sufficiently. This inconsistency in standards, which has been reported in other areas of big data research (Samuel et al., 2019), led to some researchers “*cheating, I would say, to make your models seem more accurate than they are*,*”* that is, because some journals did not require researchers to validate their models for publication, researchers could pick and choose where they published their research to avoid validating their models responsibly or appropriately. As computer scientist, interviewee 9, explained (and providing an example of the divisions between computer science and other more health-related disciplines):

if we submit a paper to some public medicine journal…the reviewer asks this question, where did you get it, how is it informed…But when you submit a paper to some other general engineering or computer science journals, they won't ask you these things, which means they completely ignore this issue (interviewee 9)

Furthermore, this lack of standards was compounded by a perceived paradoxical discourse from journals and funding bodies, which, on the one hand, were seen to call for more ethical research, but on the other hand, only rewarded novelty and innovation. In the extracts below, we can see how interviewees struggled to validate their AI models responsibly because both journal editors and funding bodies wanted to publish and fund research that was new and exciting. In spite of the calls for “responsible AI,” it was hard, explained interviewees, to get funding for the ethical aspects of their research:

it's tough sometimes, because…funding agencies are really about novelty, so they are really happy when you come up with something new. When you talk about validation…it's a little less easy to get funding…to conduct this work (interviewee 16)

in order to succeed in my career, I need to publish, and I find that reviewers are pushing me to say good things about the method…I find a lot of people in the field are successful because they ignore the failings of their methods (interviewee 15).

### Mapping Exercise

**Figure 1.**
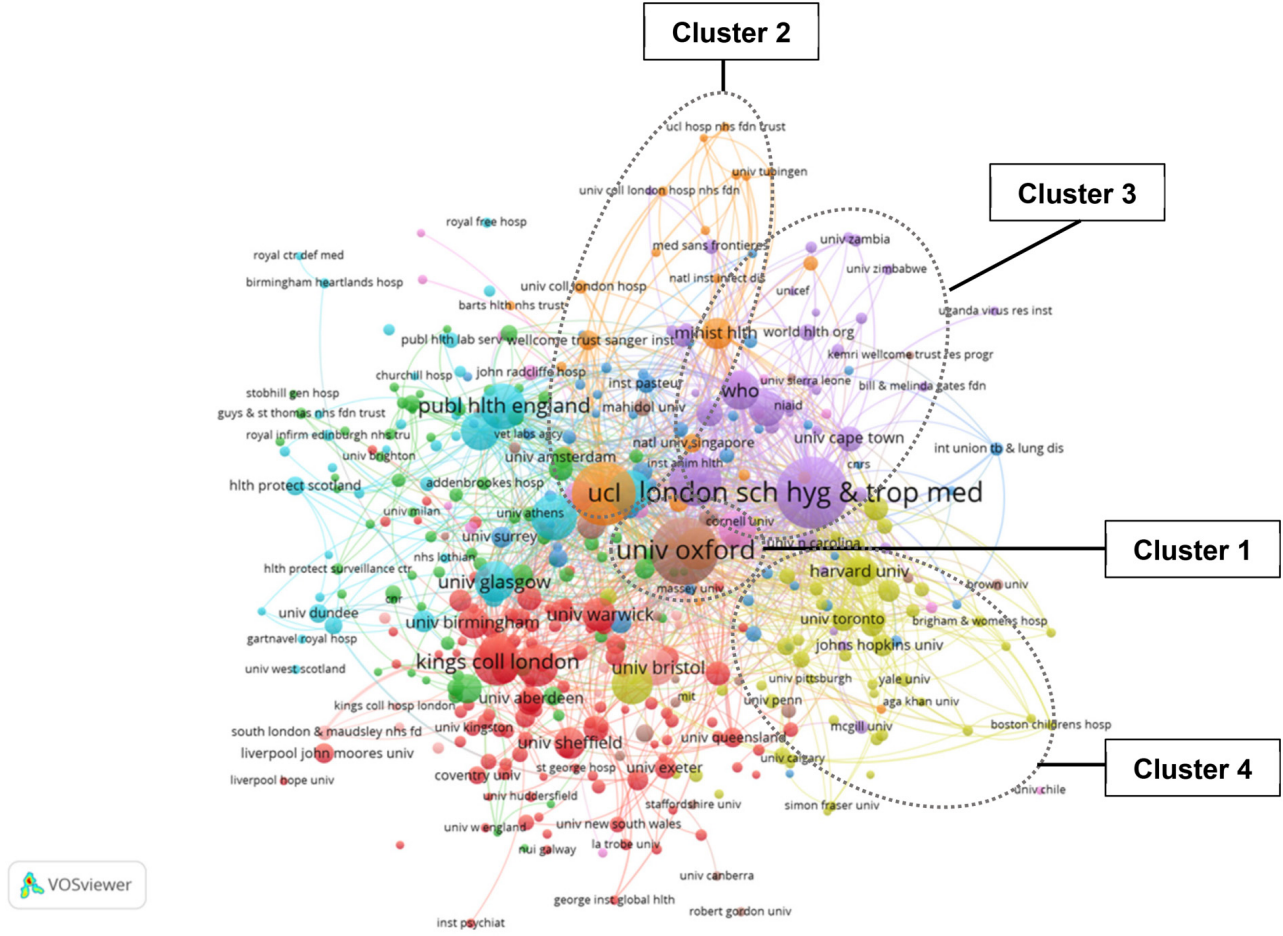
Cocitation map of affiliated organizations with a minimum of 10 papers with at least 10 citations.

A cocitation map of author affiliations with at least 10 publications with 10 citations each was constructed using VOSviewer 1.6.14^6^ and is shown in Figure 1. The results show seven distinct main clusters with the University of Oxford (Cluster 1) cluster most centrally located. Other clusters from the U.K. sample include University College London (UCL, Cluster 2) and the London School of Hygiene and Tropical Medicine (Cluster 3) as other dominant clusters. Cluster 3 is the only cluster that includes connected affiliations with international, nonprofit organizations such as the World Health Organization. The U.S./Canada cluster (Cluster 4) is peripherally located with weak ties located in the central Clusters 1–3.

For each cluster, hospital affiliates were all peripherally located, whereas universities and academic organizations were more centrally located, demonstrating the prominence of research organizations in generating research as compared to stakeholder affiliations. This could be related to the access that hospitals have to patient data, and a unique Ethics Ecosystem (Samuel et al., 2019) that governs access to patient data, or the formal relationships academics hold with hospitals as nonacademic stakeholders in the research, and how a partnership is seen to influence the ability for findings, specifically AI tools, to be put into practice beyond academia as part of the science-society contract (Gibbons, 1994).

## Discussion

In this paper, we have reported how our population health AI researcher interviewees tended to separate their responsibilities related to the ethical practice of research from the ethical use of research. Specifically, interviewees constructed their discussions around research ethics as procedural, routine, and comprising a key aspect of the HEI ethics governance structure, whereas their discussions on the ethical use of research were more commonly framed as less tangible and more lacking in effective oversight. This was echoed in the visualization of affiliation networks where academic organizations were more centrally located to each cluster than the affiliated societal partners (usually hospitals). This suggests an institutional divide to the regulation and ethical use of patient-based AI data for practical (societal partner) and research (academic partner) use, where partnerships with health stakeholders are made for pragmatic reasons such as to enable access to the data and/or to increase the societal relevance of the resulting research. Differing incentive structures surrounding successful publication could also result in collaborations between researchers and stakeholders that are more pragmatic (governing the access to health data as a primary goal) rather than intellectual. However, this is unlikely to be the primary explanation as researchers in the United Kingdom affiliated to stakeholder organizations are likely to be subject to the same performative expectations as researchers solely affiliated with universities.

The separation between the ethical practice of research and research use is not necessarily problematic in and of itself. However, the differentiation did permit another issue to be exposed. That is, while the governance of research ethics is tightly regulated, the ethics governance of research use is not. This is problematic if researchers are being called upon to consider the ethics of the societal impact of the research, and take responsibility for this, because, as we saw in our findings, it leaves them with little way to enact such responsibilities. This lack of an ethics governance structure to oversee and respond to issues associated with research use can be seen as a manifestation of the functioning of the Ethics Ecosystem, in which the purview of RECs is primarily focused on issues of research ethics, leaving little space and less oversight for considerations related to the ethical use of research. This is problematic because the Ethics Ecosystem can govern research practice from inception to publication through its actors’ shared understanding of research ethics, however, once research is published and disseminated, it is subject to little ethics oversight; and the research can potentially become distorted, misrepresented, and/or used inappropriately (Brundage, 2016). As we discuss further below, the Ethics Ecosystem needs to be modified to allow for a body to support ethics decision making at the level of research use. Questions remain regarding whether RECs should or even could fulfill this role, or whether such governance should be overseen by another body (Samuel & Derrick, 2020; Ferretti et al., forthcoming).

Our findings highlight other issues within the Ethics Ecosystem, particularly around the lack of promotion and incentives for responsible and appropriate research use. As we have described previously, Ethics Ecosystem actors need to participate equally in the promotion, evaluation, and enforcement of a shared understanding of ethically responsible research behavior to keep the Ethics Ecosystem in equilibrium. There is a risk that the system can become imbalanced when this shared understanding breaks down (Samuel et al., 2019). Our findings have highlighted clear examples of disequilibrium—both at the level of the REC with their narrow focus on data governance (see below), as well as more broadly at the level of funding bodies’ and journals’ focus on research novelty at the expense of the more mundane, but ethically responsible repetition, verification, and validation of research. As researchers try to take more responsibility for both their research and research use, researchers will only be able to enact this responsibility if their vision of ethics best practice is shared *and* practiced among all “Ethics Ecosystem” actors, including funding bodies and journals, and if their epistemic responsibilities are valued by institutions.

Finally, the REC focus on data governance issues that was seen in our interview findings warrants further attention. The fact that most discussions focused on privacy was problematic and led to a narrowing of ethics and responsibility debates being perpetuated throughout the Ethics Ecosystem, often at the expense of other ethical issues such as questions around justice and fairness (McQuillan, 2018). This can be analogized to a similar situation—that of consent. Here a disproportionate focus on this concept, along with the importance RECs place on consent forms and participant information sheets, has led some way to establishing how research ethics is defined (Whelan, 2018), that is, with consent often being viewed (sometimes incorrectly) as a proxy for ethics best practice, or in some cases, as an ethics panacea (Corrigan, 2003).

In conclusion, we have shown how our interviewees separated their responsibilities related to the ethical practice of research from the ethical use of research. We note that given the small sample size further work needs to explore the generalizability of our findings and to consider how far this is particular to AI health research. Further research also needs to explore the relevance of our findings beyond the population health AI field to AI research more broadly, and also other data-driven research. Below we highlight relevant best practices that emerge from our findings, a future research agenda, and the educational implications.

## Best Practice

If we do want researchers to be reflexive about the ethical, social, and political impacts of their population health AI research (and one could hypothesize, data-driven research more generally), the current Ethics Ecosystem and HEI ethics governance framework need modification. Relying on researchers own perceived responsibilities is appropriate, but as we described above, only if these responsibilities are shared *and* supported across all other actors in the Ethics Ecosystem, including RECs, funding bodies, and journal editors—both locally as well as globally (ÓhÉigeartaigh et al., 2020). We argue that best practice should require this shared and supported set of responsibilities.

## Research Agenda

We and others have previously called upon HEIs to take a more active role in driving ex-post population health-related AI research ethics by building infrastructure and culture of open (or tiered) access to data sets, workflows, and algorithms to allow easier validation of AI models (Gasser, 2017; see also Herrick & Sarewitz, 2000); and for algorithms to be recognized and governed as research outputs in much the same way as peer-reviewed articles (The hidden REF, 2020). We have also called for an additional Ethics Ecosystem actor to govern ex-post research review for population health-related AI research to synergize thinking around the ethics of research and its impact (Samuel & Derrick, 2020). We argue that, in light of our findings, such calls be taken seriously in future research agendas.

## Educational Implications

Scholars have argued that population health research ethics needs to open-up; it needs to revolve around a public health ethics framework, focusing on justice, promoting population health, and reducing inequalities, rather than protecting individuals from potential research harm (Ballantyne, 2019; Xafis et al., 2019). We support this. Although, as our findings on the separation of ethics practices of research and research use have highlighted, difficulties remain regarding how to responsibly assess such ideals that relate more to the ethics of research use, especially since these are not envisioned to be under the purview of RECs. We do not have an answer for this here, but emphasize the need for researchers, RECs, and other actors of the Ethics Ecosystem to engage in discussions about these inconsistencies, to promote education and awareness. Scholars have argued that some researchers have been led to believe that they do not always actually impact on human decision making in the field of AI and machine learning (Hagendorff, 2020, p. 1). While our research findings did not necessarily show this (perhaps because of the self-selected criteria), here, the role of education is even more vital.

## Notes

1. Notwithstanding the discourse on ethics washing (see, for example, Samuel & Farsides, 2018; Wagner, 2018).

2. See, for example, https://www.leverhulme.ac.uk/leverhulme-research-centres/leverhulme-centre-future-intelligence.

3. https://www.computerweekly.com/news/252470371/UK-artificial-intelligence-investment-reaches-record-levels.

4. Also see http://www.cihr-irsc.gc.ca/e/51204.html and https://www.gov.uk/government/organisations/centre-for-data-ethics-and-innovation.

5. Interviewees emphasized that because of the complexity of AI research, transparency must not be confused with explainability, and while we can move toward transparent AI, it would be unrealistic to make AI explainable, nor to expect nonspecialists to understand how the algorithms worked. Rather, explained interviewee 6, the algorithm only needs to be open to scrutiny. There is a move toward literacy.

6. Downloaded from https://www.vosviewer.com/ on January 7, 2020.
